# Antidiarrheal activity of flowers of *Ixora Coccinea* Linn. in rats

**DOI:** 10.4103/0975-9476.74422

**Published:** 2010

**Authors:** Yasmeen Maniyar, Prabhu Bhixavatimath, N. V. Agashikar

**Affiliations:** *Department of Pharmacology, S. Nijalingappa Medical College and H.S.K Hospital and Research Centre, Navanagar, Bagalkot, Karnataka, India*; 1 *Department of Pharmacology, Jawaharlal Nehru Medical College, Belgaum, India*

**Keywords:** Antidiarrheal activity, Castor oil induced diarrhea, enteropooling method, Ixora coccinea linn, Small intestinal transit, Traditional medicine

## Abstract

*Ixora coccinea* Linn (Rubiaceae), a small shrub cultivated throughout India, has been reported to possess a number of medicinal properties. It has traditionally been used for the treatment of diarrhea and dysentery. However the claims of Ayurveda have to be validated by suitable experimental models. The present study was therefore undertaken to evaluate the effect of aqueous extract of *I. coccinea* for its antidiarrheal potential against several experimental models of diarrhea in albino Wistar rats. Here, we report the effects of aqueous extracts of flowers of *I. coccinea* in the castor oil induced diarrhea model. The gastrointestinal transit rate was expressed as the percentage of the longest distance traversed by charcoal divided by the total length of the small intestine. Weight and volume of intestinal content induced by castor oil were studied by the enteropooling method. Loperamide was used as a positive control. The plant-extract showed significant (*P*<0.001) inhibitor activity against castor oil induced diarrhea and castor oil induced enteropooling in rats at the dose of 400 mg/kg. There was also significant reduction in gastrointestinal motility in the charcoal meal test. Results obtained in this study substantiate the antidiarrheal effect of the aqueous extract and its use by traditional practitioners in the treatment of diarrhea.

## INTRODUCTION

In developing countries, diarrhea continues to be one of the leading causes of mortality and morbidity in children less than 5 years old. According to World Health Report, diarrhea is the cause of 3.3% of all deaths. Worldwide distribution of diarrhea accounts for more than 5-8 million deaths each year in children. The incidence of diarrheal disease still remains high despite the effort by many government and international organizations to reduce it. Use of traditional medicines to combat the consequences of diarrhea has been emphasized by WHO in its Diarrhoea Control Programme.[1–6] It is therefore important to identify and evaluate available natural drugs as alternatives to current antidiarrheal drugs, which are not always free from adverse effects. Several studies have shown the beneficial effects of a number of medicinal plants used traditionally in the treatment of diarrheal disease, one such being *Ixora coccinea*.[7–13]

*I. coccinea* Linn. is a small shrub cultivated throughout India. (Flame of Woods in English, Rangan in Hindi and Bengali, Kisukare in Kannada.) Roots and flowers are used in dysentery, dysmenorrhea, leucorrhoea, hemoptysis, and catarrhal bronchitis. Leaves are used in diarrhea. Roots are also used in hiccup, nausea, loss of appetite and externally for the treatment of sores, eczema, chronic ulcers. Roots contain aromatic acrid oil, tannin, fatty acids. Leaves yield flavonols, kaemferol, quercetin, proanthrocyanidines, phenolic acids, and ferulic acids. Flowers yield cyanidins, flaconboides, and cooling material related to quercitin. Roots ground into pulp, mixed with water and as tincter are used for diarrhea and dysentery.[14–21] However, scientific evidence to verify these claims is limited.

The present study was undertaken to evaluate the antidiarrheal activity of extracts of *I. coccinea* Linn flowers against experimentally induced diarrhea.

## MATERIAL AND METHODS

### Plant material

The flowers used in the study were collected in Karnataka’s Belgaum district in the month of November-December, 2007 and authenticated by Prof: A. P. Kore, Department of Botany, R.L.S. College Belgaum. The voucher specimen (number 00545) was deposited in the departmental herbarium for further references. The flowers were shade dried for a period of 4 weeks after which they were finely powdered. Cold flower extracts were prepared according to the method described by Rawlins.[22] The powder was dissolved in water in the ratio of 1:3 (250 mg of powder in 750 ml of distilled water), and shaken three to four times a day for a period of 7 days.[23] After filtration, the filtrate was concentrated and dried under reduced pressure. The extract was brown in color, semisolid form, with a yield of 18.6% (w/w). The extract it was stored in desiccators until use.

### Phytochemical screening

The freshly prepared extract was subjected to standard phytochemical screening tests for various constituents:[24] alkaloids, glycosoids, tannins, saponins, sterols, and flavanoids.

### Animals used

Albino Wistar rats weighing 150-200 g of both sexes were used. They were housed in standard polypropylene cages, at room temperature (24 ± 2°C) and exposed to a 12:12 h light and dark cycle. The rats were fed on a standard diet (Gold Mogr Lipton India Ltd.) and water ad libitum. The study protocol was approved by the institutional animal ethical committee (Ethical committee IAEC-reg.no.627/02/a CPCSEA) of Jawaharlal Nehru Medical College, Belgaum.

### Castor oil induced diarrhea

The study employed the method described by Niemegeers *et al*.[25] The rasts were fasted for 24 h before the test with free access to water, and divided into five groups of six animals each. Diarrhea was induced by administering 1 ml of castor oil orally. Group I treated as control (2 ml/kg, ip saline), group II received loperamide (5 mg/kg) served as standard, and group III-V received extract (100, 200, and 400 mg/kg ip) 1 h before castor oil administration. Consistency of fecal matter, numbers of both wet and dry diarrheal droppings were counted every hour for a period of 4 h.

### Castor oil induced enteropooling

Intraluminal fluid accumulation was determined by the method of Robert *et al*. and Dicarlo *et al*.[26,27] The rats were divided into five groups of six animals each, were fasted overnight, but allowed free access to water. Group I treated as control (2 ml/kg ip saline), group II received loperamide (5 mg/kg ip) treated as standard. Groups III-V received extract (100, 200, and 400 mg/kg ip). Then 1 h later, 2 ml of castor oil orally was administered to these groups for induction of diarrhea. Two hours later, the rats were sacrificed, and the small intestine ligated at both the pyloric sphincter and the ileocaecal junction and dissected. The small intestine was weighed and its contents collected by milking into a graduated tube allowing the volume to be measured; the intestine was then reweighed and the difference between full and empty weights calculated.

### Small intestinal transit

The method described by Jansen and Jageneau was used.[28] Rats were fasted for 18 h and divided into six groups of six animals in each. Group I received normal saline 2 ml/kg orally. Group II received 2 ml of castor oil orally with 2 ml/kg normal saline intraperitoneally. Group III received loperamide (5 mg/kg, ip), group IV-VI received 100, 200, and 400 mg/kg intraperitoneally of plant extract, 1 h before administration of castor oil. One milliliter of marker (10% charcoal suspension in 5% gum acacia) was administered orally 1 h after castor oil treatment. The rats were sacrificed after 1 h and the distance traveled by the charcoal meal from pylorus to caecum was measured and expressed as the percentage of the whole length of the intestine.

### Statistical analysis

Experimental results are represented as mean ± SE (standard error of mean). Student’s t test was used for the evaluation of data.

## RESULTS

### Phytochemical screening

Results of preliminary phytochemical screening of the aqueous extracts revealed the presence of alkaloids, flavonoids, tannins, glycosoides, and absence of saponins and sterols.

### Castor oil induced diarrhea

Diarrhea was apparent in all the animals of control group 30 min after administration of castor oil, for the next 4 h. This was largely eliminated by intraperitoneal injection of loperamide, 5 mg/kg (48.12%) [Table 1]. The effect of the extract was not as potent as loperamide in the dose of 100 mg/kg, but in the doses 200 and 400 mg/kg, the extract produced a significant dose-dependent reduction in the number of defecations over 4 hours (*P* < 0.001).

**Table 1:** Effect of aqueous extract of flowers of *I. coccinea* on castor oil induced diarrhea in rats

Treatment	Mean defecation in 4 hr (g/kg body wt.)	% of Inhibition of defecation
Castor oil + saline (2 ml/kg ip)	23.51 ± 0.349	0
Castor oil + loperamide (5 mg/kg ip)	12.2 0± 1.7**	48.12
Castor oil + extract (100 mg/kg ip)	21.2 ± 0.59*	9.82
Castor oil + extract (200 mg/kg ip)	16.65±0.21**	29.17
Castor oil + extract (400 mg/kg ip)	11.05± 0.28**	52.99

Extract was administrated ip 1 hr before castor oil administration. Values are expressed as mean ± SEM from the experiments.

* *P*<0.01

** *P*<0.001 when compared with castor oil + saline treated group

### Castor oil induced enteropooling

Castor oil caused accumulation of water and electrolytes in the intestinal loop. Treatment with extract (100, 200, and 400 mg/kg) produced a significant, dose-dependent reduction in intestinal weight and volume [Table 2]. Significant results (P < 0.001) were observed at doses of 200 and 400 mg/ kg

**Table 2:** Effect of aqueous extract of flowers of *I. coccinea* on castor oil induced enteropooling in rats

Treatment	Wt. of intestinal content (g)	% Inhibition of weight of intestinal content
Castor oil + saline (2 ml/kg ip)	2.41 ± 0.12	----
Castor oil + loperamide (5 mg/kg ip)	1.61± 0.12**	32.78
Castor oil + extract (100 mg/kg ip)	1.58 ± 0.55*	34.43
Castor oil + extract (200 mg/kg ip)	1.09±0.12**	54.77
Castor oil + extract (400 mg/kg ip)	1.12± 1.003**	56.67

Extract was administrated ip 1 hr before castor oil administration. Values are expressed as mean ± SEM from the experiments.

* *P*<0.01

** *P*<0.001 when compared with castor oil + saline treated group

### Small intestinal transit

The aqueous extract of *I. coccinea* significantly decreased the propulsion of the charcoal meal through the gastrointestinal tract compared to the control group. Loperamide (5 mg/kg) produced a marked decrease in the propulsive movement and intestinal length traveled by the charcoal [Table 3].

**Table 3:** Effect of extract of flowers of *I. coccinea* on castor oil induced small intestinal transit in rats

Treatment	Total length of intestine	Distance travelled by Marker	% of Intestinal transit
Saline (2 ml/kg po)	85.8 ± 2.68	82.81 ± 3.01	96.45
Castor oil + saline (2 ml/kg ip)	78.21 ± 2.92	76.68 ± 2.76	90.37
Castor oil + loperamide (5 mg/kg ip)	94.91± 2.84	38.58 ± 2.76**	40.65
Castor oil + extract (100 mg/kg ip)	71.51 ± 4.82	73.88 ± 4.36	70.48
Castor oil + extract (200 mg/kg ip)	83.83 ± 3.04	63.23 ± 1.78**	75.58
Castor oil + extract (400 mg/kg ip)	86.06 ± 2.21	59.1 ± 3.34**	68.95

Extract was administrated ip 1 h before castor oil administration. Values are expressed as mean ± SEM from the experiments.

* *P*<0.01

** *P*<0.001 when compared with castor oil + saline treated group

## DISCUSSION AND CONCLUSION

Results of this study suggest that aqueous extracts of flowers of *I. coccinea* in graded doses of 100, 200, and 400 mg/kg body weight reduced diarrhea by inhibiting intestinal motility, intestinal fluid accumulation, significantly reducing the frequency of defecation. This justifies folk medicine’s use of aqueous extract of *I. coccinea* Linn.

Castor oil is known to produce changes in intestinal mucosal permeability to electrolyte and water leading to diarrhea.[29,30] Antidiarrheal activity of these extracts may be attributable to one of the following mechanisms:

- The extract may increase the reabsorption of NaCl and water by decreasing the intestinal motility by charcoal meal.
- The presence of tannates in the extract may make the intestinal mucosa more resistant and reduce the secretion.[31,32] Tannic acid and tannins are water-soluble polyphenols that are present in many plants.[33]
- Liberation of recinoleic acid by castor oil results in irritation and inflammation of intestinal mucosa leading to release of prostaglandins.[34,35] The extract may reduce prostaglandin secretion.
- Flavonoids and alkaloids are known to inhibit release of autocoids and prostaglandin, thereby inhibiting secretion induced by castor oil.[36,37] Phytochemical analysis of aqueous extract of *I. coccinea* showed the presence of flavonoids, alkaloids, and tannins. Antidiarrheal and antidysenteric properties of medicinal plants were found to be due to tannins, alkaloids, saponins, flavonoids, sterols, and reducing sugars.[38] Sesquiterpenes, diterpenes, terpenes, flavonoids, and terpenoid derivatives are known for inhibiting release of autocoids and prostaglandins, thereby inhibiting the motility and secretion induced by castor oil.[39,40] Loperamide, a synthetic opiate analogue, regulates the gastrointestinal tract by inhibiting its propulsive motor activity, predominantly in the jejunum and this effect is partially inhibited by opiate antagonists. Loperamide is also reported to reduce colonic rate of flow and consequently increase colonic water absorption, but it does not have any effect on colonic motility.[41]

The study has intentionally been undertaken using a crude aqueous extract as it is our belief that the different biological activities assessed herein may not be due to a single constituent. This has also been highlighted by Mayer Manga *et al*,,[42] who have stated that the crude extracts contain several compounds acting on different mechanisms. In addition interplay of constituents in the crude extract may result in better activity due to synergism or lead to decrease in toxicity; it is possible that pure compounds do not behave in the same manner as natural extracts.[43,44]

To conclude, the present study supports claims by traditional medical practitioners about the use of aqueous extracts of *I. coccinea* Linn. in the treatment of diarrhea. The study included multiple bioassays covering the entire spectrum of activities that can provide more reliable evaluation of the plant’s biological efficacy. The active constituent(s) responsible for antidiarrheal activity remain to be identified; further studies are required to understand the mechanism of action of its antidiarraheal activity.
